# Detection of African Swine Fever Virus and Blood Meals of Porcine Origin in Hematophagous Insects Collected Adjacent to a High-Biosecurity Pig Farm in Lithuania; A Smoking Gun?

**DOI:** 10.3390/v15061255

**Published:** 2023-05-26

**Authors:** Ann Sofie Olesen, Jonno Jorn Stelder, Kirsten Tjørnehøj, Camille Melissa Johnston, Louise Lohse, Lene Jung Kjær, Anette Ella Boklund, Anette Bøtner, Graham J. Belsham, René Bødker, Thomas Bruun Rasmussen

**Affiliations:** 1 Section for Veterinary Virology, Statens Serum Institut, Artillerivej 5, DK-2300 Copenhagen, Denmark; kitj@ssi.dk (K.T.); camj@ssi.dk (C.M.J.); lolo@ssi.dk (L.L.); tbru@ssi.dk (T.B.R.); 2 Section for Animal Welfare and Disease Control, Department of Veterinary and Animal Sciences, University of Copenhagen, Grønnegårdsvej 8, DK-1870 Frederiksberg C, Denmark; jonno.stelder@sund.ku.dk (J.J.S.); lenju@sund.ku.dk (L.J.K.); anebo@sund.ku.dk (A.E.B.); rebo@sund.ku.dk (R.B.); 3 Section for Veterinary Clinical Microbiology, Department of Veterinary and Animal Sciences, University of Copenhagen, Stigbøjlen 4, DK-1870 Frederiksberg C, Denmark; aneb@sund.ku.dk (A.B.); grbe@sund.ku.dk (G.J.B.)

**Keywords:** ASF, hematophagous insects, high-biosecurity farm, virus transmission, vector, virus introduction

## Abstract

A seasonal trend of African swine fever (ASF) outbreaks in domestic pig farms has been observed in affected regions of Eastern Europe. Most outbreaks have been observed during the warmer summer months, coinciding with the seasonal activity pattern of blood-feeding insects. These insects may offer a route for introduction of the ASF virus (ASFV) into domestic pig herds. In this study, insects (hematophagous flies) collected outside the buildings of a domestic pig farm, without ASFV-infected pigs, were analyzed for the presence of the virus. Using qPCR, ASFV DNA was detected in six insect pools; in four of these pools, DNA from suid blood was also identified. This detection coincided with ASFV being reported in the wild boar population within a 10 km radius of the pig farm. These findings show that blood from ASFV-infected suids was present within hematophagous flies on the premises of a pig farm without infected animals and support the hypothesis that blood-feeding insects can potentially transport the virus from wild boars into domestic pig farms.

## 1. Introduction

African swine fever (ASF) is a severe hemorrhagic disease affecting members of the suidae family. The disease is caused by African swine fever virus (ASFV), a large double-stranded DNA virus. This virus is the sole member of the *Asfarviridae* family (genus *Asfivirus*) and the only known DNA arbovirus [1]. Following its transfer from Africa into Georgia in 2007, the virus spread into Russia and further into Eastern Europe in 2014 [2]. ASFV infection has reached pandemic proportions, now affecting countries worldwide [3] including Haiti and the Dominican Republic in the west, across Europe to Russia, China and the Philippines in the east. In affected countries within Eastern Europe, a seasonal trend of ASF outbreaks in domestic pig farms has been observed—with disease outbreaks peaking during the warmer summer months [4]. This peak coincides with the seasonal pattern of increased activity by hematophagous (blood-feeding) insects such as mosquitos, biting midges, horseflies and stable flies [4]. ASFV DNA has been detected in hematophagous insects on outbreak farms [5,6,7]. However, it remains unclear whether the ASFV DNA derived from insect blood meals was obtained from pigs on the outbreak farm or was introduced by infected domestic pigs or wild boar sources outside the outbreak farm [8]. It is possible, however, that hematophagous insects could circumvent biosecurity measures at high-biosecurity pig farms after feeding on an ASFV-infected wild boar or domestic pig present within other pig farms in the surrounding area and afterwards introduce the virus into new farms. Recently, a field study in Lithuania sought to identify (1) which insects were present in the vicinity of two high-biosecurity pig farms, (2) which insects attempted to enter these farms and (3) the presence and source of blood meals within these insects. It was demonstrated that hematophagous insects could carry blood meals taken from outside sources into these two high-biosecurity farms [8]. In addition, ASFV DNA was detected in a single pool of insects—more specifically, *Haematopota* spp.—caught in a trap just outside one of the farms, which did not have ASFV-infected animals [8]. In the light of this finding, we decided to screen additional pools of hematophagous flies, collected during the same study at that farm, for the presence of ASFV DNA and, furthermore, to try to identify the source of the mammalian blood in the pools.

## 2. Materials and Methods

The analyzed insects (hematophagous flies) were collected in 2020 on the edge of a high-biosecurity pig farm located in the southern Šiauliai County, Lithuania. The farm experienced an outbreak of ASF in 2018 [8], which was followed by the culling of all pigs in the herd according to EU law (https://eur-lex.europa.eu/legal-content/EN/TXT/?uri=celex%3A32002L0060, accessed on 16 May 2023). It is situated in a rural region with a forested area on its northern side and consists of 10 buildings (20 stables) with ~18.000 pigs, including 1250 sows. Hematophagous flies were collected in five H-traps (labelled traps A, B, C, D and E) located outside the stables but within the inner fence of the farm [8]. The collection dates were 12 August, 19 August and 16 September 2020 (Table 1), and the insects represent weekly collections from four of the traps (traps B, C, D and E, Table 1). The insects were preserved in 66% ethanol, shipped to the laboratory and then sorted prior to analysis as described previously [8]. Briefly, hematophagous flies were pooled according to location, date, trap number and genus. In addition, they were pooled according to their size, meaning that the smaller hematophagous flies, such as *Haematopota* spp. and *S. calcitrans,* were pooled in groups of five, while larger hematophagous flies, such as *Tabanus* spp., were pooled in groups of only 2 or tested individually (Table 1). MagNA Pure Lysis/Binding Buffer (Roche, Basel, Switzerland) (1 mL) and two 3 mm stainless steel beads (Dejay Distribution Ldt., Launceston, UK) were added to each pool and the samples were homogenized for 3 min at 25 Hz using a TissueLyser II (Qiagen, Hilden, Germany). The homogenates were centrifuged for 2 min at 10,000 RCF and supernatants collected for DNA extraction, which was performed using a MagNA Pure 96 system (Roche) [9]. DNA samples extracted from 48 pools, consisting of 195 insects in total (167 *Haematopota* spp., 9 *Tabanus* spp. and 19 *S. calcitrans*; Table 1), were tested separately for the presence of ASFV DNA using the quantitative PCR (qPCR) assay described by Tignon et al. [10]. Samples in which ASFV DNA was detected were then tested in triplicate using the Virotype ASFV 2.0 (Indical Bioscience, Leipzig, Germany) assay according to the manufacturer’s instructions to confirm the results. In both assays, a positive result was defined as giving a Cq value, at which FAM dye emission appeared above background, below 42.

Pools in which ASFV DNA was detected were also tested for the presence of a blood meal of porcine origin using a TaqMan assay designed to detect the mitochondrial cytochrome b gene from suidae [11]. A positive result (suid blood present) in this qPCR was defined as having a Cq value below 35. The ASFV DNA and suid mitochondrial DNA assays were performed on the CFX Opus Real-Time PCR System (Biorad, Hercules, CA, USA). The samples were further tested for the presence and source of a mammalian blood meal (by detection of a mammalian mitochondrial cytochrome b gene) using a qPCR followed by Sanger sequencing of the 127 bp amplicon using primer sequences slightly modified from Andrejevic et al. [12], as previously described [8,13]. Sequencing results were analyzed using Sequence Scanner Software v1.0 (Applied BioSystems, Waltham, MA, USA) and the blood meal source identified using BLAST [14].

Finally, Illumina (San Diego, CA, USA) MiSeq sequencing was performed on the extracted DNA as described previously [15], except for the up-concentration step. Raw reads were trimmed using AdapterRemoval v. 2.3.3 [16]. The trimmed reads were mapped to near or complete full-length mitochondrial sequences from *S. calcitrans* (DQ533708.1), *Haematopota spp.* (MT483662.1, MT584146.1) and *Tabanus spp.* (NC_062705.1, NC_068061.1), as well as mitochondrial RefSeq sequences from *Bos taurus* (NC_006853.1) and *Sus scrofa* (NC_000845.1) using BWA-MEM [17], then filtered for duplicated reads and mapping quality 60 using Picard MarkDuplicates v.2.27.4 [18] and SAMtools [19], respectively. Reads mapping to *S. calcitrans* and *Bos taurus* mitochondria from pool 79 were BLASTed to the NCBI database [14] using Megablast in Geneious 2023.0.4 (Geneious, Biomatters INC., Boston, MA, USA to confirm their origin.

## 3. Results

Screening of these 48 pools using the assay described by Tignon et al. [10] detected ASFV DNA in six pools (Table 2). Using the ASFV Virotype 2.0 (Indical Bioscience) assay, the presence of ASFV DNA was confirmed in four of these six samples (pools 11, 12, 16 and 18). These positive pools either contained five *Haematopota* (pools 11 and 12) or two *Tabanus* (pools 16 and 18), respectively, and were all collected from trap B on 12 August 2020. In the two remaining samples (pools 39 and 79), not all qPCR reactions yielded a positive result. These insect pools contained five *Haematopota* collected in trap E on 12 August 2020 (pool 39) or five *Stomoxys* collected in trap E on 16 September 2020 (pool 79) (Table 2). Another 33 pools, obtained from the H-traps outside the same farm, were tested previously as described in [8]. ASFV DNA was detected in one of those pools (pool 23) containing five *Haematopota* spp. caught in trap D and collected on 12 August 2020 (Table 2).

The six pools, in which ASFV DNA was detected, were analyzed for the presence of porcine blood (by detection of the mitochondrial cytochrome b gene from *suidae*). When tested in triplicate, the samples yielded no Cq-value or a Cq-value above the threshold of 35 (values obtained were from 38.0 to 40.7). In contrast, using an assay designed to detect mammalian blood from a range of species, Cq-values were much lower (in the range of 18.3–31.3). Sanger sequencing of the PCR products revealed that the DNA within these pools mapped to either *Sus scrofa* or *Bos taurus* mitochondrial sequences in the BLAST database (Table 2). Using Ilumina sequencing, one pool (pool 79) provided a sufficient number of mapped reads to support the visual identification of the insects within the pool and the host blood source identified using Sanger sequencing of the PCR products. From this pool, more than 5900 reads mapped to *S. calcitrans* (DQ533708.1) while 220 reads mapped to *Bos taurus* mitochondrial DNA (NC_006853.1) (Table 3). The mapped reads were distributed across the references and were BLASTed [14], which resulted in 70.1% and 64.1% having *S. calcitrans* and *Bos taurus* as their first hit, respectively, using an Identity cut-off of 99% and E-value of 1 × 10^−9^ In the remaining five pools, the numbers of reads were too low to make any certain conclusions from the DNA present within them (Table 3).

## 4. Discussion

In the current study, we demonstrated the presence of ASFV DNA in hematophagous flies (*Haematopota* spp., *Tabanus* spp. or *Stomoxys*) caught inside the perimeter fences (that exclude all larger mammals) of a high-biosecurity pig farm in Lithuania that did not report ASFV-infected pigs during the study period in 2020. Stelder et al. [8] found that semi-/fully engorged insects are attracted to high-biosecurity pig farms and that flies of the same genera (i.e., *Haematopota* spp., *Tabanus* spp. and *Stomoxys* spp.) try to enter the farms through window openings. Furthermore, these insects can carry blood meals from outside sources, e.g., from infected pigs or wild boar. Thus, these findings indicate that hematophagous flies carrying ASFV can potentially act as a source of virus introduction into pig farms. Even though ASFV DNA was present in flies circulating around the farm included in this study, it had not experienced an outbreak of the disease since 2018. At the farm, strict biosecurity measures were implemented (including insect control measures such as mosquito nets on all windows and H-traps around the farm [8]).

The four pools, in which the viral DNA was detected in each of the qPCRs (pools 11, 12, 16 and 18), all originated from a weekly collection of *Haematopota* spp. or *Tabanus* spp. from trap B emptied on 12 August 2020. A weekly collection from the separate trap D, on the same collection date, was the source of the one pool reported to contain ASFV DNA previously [8]. The borderline positive pools were both from weekly collections from trap E, either on the same date (pool 39 consisting of *Haematopota* spp. on 12 August 2020) or from a later collection (pool 79 consisting of *S. calcitrans* on 16 September 2020). During the study period, horse flies had the highest abundance in the H-traps, while *S. calcitrans* appeared (sporadically) in these traps at the end of the collection period [8]. This could explain why the pools containing ASFV DNA from mid-August consisted of *Haemotopotae* spp. and *Tabanus* spp., while the pool found to contain ASFV DNA in mid-September consisted of *S. calcitrans*. According to the Lithuanian veterinary services (data extracted from state veterinary maps in December 2020), ASFV was detected in two wild boars within a radius of 10 km (8.6 km and 7.3 km) from the farm on 7 September 2020 and 8 October 2020, respectively [8]. The detection of ASFV DNA within the five insect pools collected mid-August and one pool collected mid-September indicates the presence of the virus within the area even before the first detection of the ASFV-positive wild boar at the beginning of September 2020.

The hematophagous flies within the analyzed pools had been homogenized as a pool; thus, it was not possible to determine if only one, or more, insects in the pool contained the virus. The findings of multiple pools containing ASFV DNA does, however, indicate that more than one insect carried the virus. Since the flies within four of the pools were present within the same collection, it could be speculated that several positive pools were due to outside contamination of the insects.

Sequencing, using primers designed to amplify a 127 bp region of the mammalian mitochondrial cytochrome b gene, showed that DNA of *Sus scrofa* or *Bos taurus* origin was present within the pools. As also mentioned above, more than one insect was present within each pool; so, different blood meal sources can be present within the individual pools, i.e., it is not unlikely that *Sus scrofa* blood would also be present in pools with *Bos taurus* blood but perhaps in lower volumes or in a more degraded state (e.g., in pools 18 and 79). This presumably explains the presence of ASFV DNA within a pool found to contain *Bos taurus* blood. The high Cq-values obtained using the TaqMan assay, designed to detect the mitochondrial cytochrome b gene from suidae, indicates that some of these blood meals could be in a degraded state, perhaps to different degrees. We previously observed that the suid TaqMan assay is more sensitive to degradation of the insect samples compared with the assay designed to detect the mitochondrial cytochrome b gene from mammals (unpublished results from A.S.O.). This could perhaps be attributed to the length of the amplicons produced by the two assays: 274 bp in the suid TaqMan assay and 127 bp in the mammalian assay. Degradation of the samples could also be one explanation for the fact that a sufficient number of high-quality reads was obtained just from one pool, i.e., pool 79, using Ilumina sequencing. The results from this pool do, however, show that next-generation sequencing can be applied to investigate the source of insects and blood meals in (well-preserved) insects collected in the field. It should be noted that in order to obtain only the highest-quality reads, strict parameters for read quality were applied during the data trimming steps. Without these strict parameters, reads within the range of 2–34 reads per pool were found to map to the *Sus scrofa* sequence (NC_000845.1). Without using strict quality parameters, the highest number of reads (34 reads) mapping to the *S. scrofa* reference was obtained from pool 79, which was also the pool with the highest number of overall mapped reads. Thus, in contrast to the Sanger sequencing approach, this finding indicates that next-generation sequencing can allow for identification of several blood meals within a pool of insects. *S. calcitrans* are known to feed on both cattle [20] and suids [21]; hence, detecting both types of blood meal within a pool of these insects is not unexpected.

The detection of blood of porcine origin in some of the pools in the middle of August 2020 (pools 11, 12, 16 and 39) along with the detection of ASFV DNA clearly shows that blood from at least one ASFV-infected suid was present within each of these pools. The lack of reported outbreaks in domestic pigs in the area at that time and the detection of an ASFV-infected wild boar in the beginning of September 2020 within a 10 km radius from the farm indicates that these blood meals were likely obtained from ASFV-infected wild boar.

In conclusion, the detection of ASFV DNA in pools of hematophagous flies collected within the boundaries of a pig farm without infected animals clearly demonstrates that the virus can be present in blood-feeding flies on and around pig farms and supports the hypothesis that these flies can potentially transport virus from wild boar into domestic pig farms. Such transmission seems readily preventable (e.g., using insect control measures). 

In order to assess the overall risk of this transmission of the virus into domestic pig herds, further studies regarding the viral load and presence of infectious ASFV within blood-feeding insects following feeding on an ASFV-infected suid as well as studies investigating further transmission to pigs via different routes are warranted. Some data are already available for *S. calcitrans* [22,23,24] but equivalent data are lacking for other blood feeding insects, such as *Haematopotae* spp. and *Tabanus* spp.

## Figures and Tables

**Table 1:** Overview of the 48 tested pools.

Pool Ids ^1^	No. of Insects in Each Pool	Collection Date in 2020	Trap	Genus
3–12	5	12 August	B	*Haematopota*
13	1	12 August	B	*Haematopota*
16–18	2	12 August	B	*Tabanus*
19–20	1	12 August	B	*Tabanus*
24–28	5	12 August	D	*Haematopota*
29	2	12 August	D	*Haematopota*
31	4	12 August	E	*Haematopota*
34–44	5	12 August	E	*Haematopota*
45	3	12 August	E	*Haematopota*
48	1	12 August	E	*Tabanus*
49	1	12 August	E	*Stomoxys*
56–57	1	19 August	B	*Haematopota*
60–61	5	19 August	C	*Haematopota*
68–70	5	19 August	E	*Haematopota*
79–81	5	16 September	E	*Stomoxys*
82	3	16 September	E	*Stomoxys*

^1^: The remaining pools were previously analyzed or were found to be unsuitable for analysis [8].

**Table 2:** Overview of the detection of ASFV DNA in the six pools.

Pool Ids	Insects in Pool	Collection Date in 2020	Trap	Genus	Quantitative PCR Cq Value(s) [10]	Virotype 2.0 Cq Values (Triplicate Samples)	ASFV DNA	Blood Meal Source ^2^
11	5	12 August	B	*Haematopota*	38.5	33.8/33.2/33.0	+	*Sus scrofa*
12	5	12 August	B	*Haematopota*	37.5	34.1/33.3/33.2	+	*Sus scrofa*
16	2	12 August	B	*Tabanus*	38.4	35.3/36.6/36.8	+	*Sus scrofa*
18	2	12 August	B	*Tabanus*	37.5	35.9/37.2/34.6	+	*Bos taurus*
23 ^1^	5	12 August	D	*Haematopota*	37.0/37.1/38.0	34.4/34.5/37.0/34.2/33.8/34.2/34.1/35.4/35.0	+	Not known ^3^
39	5	12 August	E	*Haematopota*	38.2	36.2/35.5/No Cq	(+)	*Sus scrofa*
79	5	16 September	E	*Stomoxys*	39.1	36.9/No Cq/No Cq	(+)	*Bos taurus*

(+) = some qPCR reactions detected ASFV DNA, + = all qPCR reactions detected ASFV. ^1^ Pool 23 was previously analyzed by Stelder et al. [8]. ^2^ Information obtained using Sanger sequencing. ^3^ The blood meal source was not identified due to degradation of the sample [8].

**Table 3:** Overview of the number of reads mapped to mitochondrial reference sequences in the six insect pools obtained by Ilumina sequencing.

Reference Sequence	Pool 11	Pool 12	Pool 16	Pool 18	Pool 39	Pool 79
*Stomoxys calcitrans* (DQ533708.1)	4	0	21	74	2	5929
*Haematopota subcylindrica* (MT483662.1)	8	4	8	22	5	0
*Haematopota pluvialis* (MT584146.1)	70	65	13	49	25	0
*Tabanus chrysurus* (NC_062705.1)	11	11	29	127	4	4
*Tabanus pleskei* (NC_068061.1)	3	0	40	107	0	0
*Sus scrofa* (NC_000845.1)	0	0	0	4	0	0
*Bos taurus* (NC_006853.1)	0	0	2	6	0	220

## Data Availability

The data used in this study are available upon reasonable request.
